# Label-Free Detection of *E. coli* O157:H7 DNA Using Light-Addressable Potentiometric Sensors with Highly Oriented ZnO Nanorod Arrays

**DOI:** 10.3390/s19245473

**Published:** 2019-12-12

**Authors:** Yulan Tian, Tao Liang, Ping Zhu, Yating Chen, Wei Chen, Liping Du, Chunsheng Wu, Ping Wang

**Affiliations:** 1 Institute of Medical Engineering, Department of Biophysics, School of Basic Medical Sciences, Xi’an Jiaotong University, Xi’an 710061, China; cnyulantian@stu.xjtu.edu.cn (Y.T.); jewel121@stu.xjtu.edu.cn (P.Z.); ytc20201011@xjtu.edu.cn (Y.C.); weixianyang@yeah.net (W.C.); duliping@xjtu.edu.cn (L.D.); 2 Biosensor National Special Laboratory, Key Laboratory for Biomedical Engineering of Ministry of Education, Department of Biomedical Engineering, Zhejiang University, Hangzhou 310027, China; cooltao@zju.edu.cn

**Keywords:** DNA biosensor, ZnO nanorod arrays, LAPS, label-free detection, *E. coli*

## Abstract

The detection of bacterial deoxyribonucleic acid (DNA) is of great significance in the quality control of food and water. In this study, a light-addressable potentiometric sensor (LAPS) deposited with highly oriented ZnO nanorod arrays (NRAs) was used for the label-free detection of single-stranded bacterial DNA (ssDNA). A functional, sensitive surface for the detection of *Escherichia coli* (*E. coli*) O157:H7 DNA was prepared by the covalent immobilization of the specific probe single-stranded DNA (ssDNA) on the LAPS surface. The functional surface was exposed to solutions containing the target *E. coli* ssDNA molecules, which allowed for the hybridization of the target ssDNA with the probe ssDNA. The surface charge changes induced by the hybridization of the probe ssDNA with the target *E. coli* ssDNA were monitored using LAPS measurements in a label-free manner. The results indicate that distinct signal changes can be registered and recorded to detect the target *E. coli* ssDNA. The lower detection limit of the target ssDNA corresponded to 1.0 × 10^2^ colony forming units (CFUs)/mL of *E. coli* O157:H7 cells. All the results demonstrate that this DNA biosensor, based on the electrostatic detection of ssDNA, provides a novel approach for the sensitive and effective detection of bacterial DNA, which has promising prospects and potential applications in the quality control of food and water.

## 1. Introduction

The detection of bacterial deoxyribonucleic acid (DNA) would have broad technological implications in areas ranging from biomedicine and the food industry, to environmental control [1,2,3,4]. For instance, the detection of *Escherichia coli* (*E. coli*) DNA is of great significance to the quality control of food and water. *E. coli* O157:H7 is one of the most dangerous foodborne pathogens, occurring in a variety of foods and water; it is able to cause hemorrhagic colitis, and leads to various symptoms, such as bloody diarrhea [5,6,7,8]. It is highly desirable to develop novel approaches for the detection of bacterial DNA in a label-free and cost-effective manner. For this reason, DNA biosensors have attracted increasing attention for their potential use in the label-free detection of bacterial DNA molecules. The cutting edge of the development of DNA biosensors is currently based on labelling strategies, that enable signal transductions and enhance the sensors’ sensitivity [9,10,11,12]. However, these approaches, while promising, suffer from inherent limitations that severely restrict their applicability. Firstly, labelling strategies require some sort of label, which increases the time and cost of DNA detection. Secondly, the labelling process can complicate the DNA-device fabrication and the DNA detection process. Furthermore, the setup of labeling approaches often requires expensive and huge instruments that limit their practical applications, especially in-field applications.

Recent progress in pushing the limits mentioned above by using label-free strategies has proven to be an alternative emerging approach for DNA detection [13,14]. The fast development of micro- and nanofabricated devices has opened up an exciting realm for the development of a new generation of label-free DNA biosensors. For instance, the label-free detection of *E. coli* O157:H7 DNA has been reported using piezoelectric sensors and electrochemical biosensors [5,15,16,17]. More recently, field-effect devices (FEDs) have offered a promising direct electrical readout for label-free DNA detection [18,19,20,21]. DNA-FEDs detect the intrinsic molecular charges of DNA molecules. These devices are unrelated to any labelling for signal transduction. They have shown tremendous promise for label-free DNA detection in a much faster, more efficient manner. The basic mechanism is based on the electrical detection of surface-charge changes caused by the hybridization of probe single-stranded DNA (ssDNA) molecules (immobilized on the sensor’s surface) with complementary target ssDNA molecules on the gate surface of the FEDs. As a result, the vast majority of DNA-FEDs are developed for the detection of ssDNA molecules. In this context, little is known about using FEDs for the direct label-free detection of bacterial ssDNA, which is a common and challenging task in many applications, such as the quality control of food and water [22,23].

The light-addressable potentiometric sensor (LAPS) is a type of FEDs. It has a light-addressable gate surface and is suitable for use as a transducer for the label-free electrical detection of DNA molecules based on their intrinsic molecular charges [21,24]. ZnO nanorods have shown promising potential in many applications, including sensors, due to their properties of having a direct wide bandgap, large exciton binding energy, and a high aspect ratio [25]. ZnO nanorods have the advantage of being highly oriented, well-structured, and being easy to prepare on substrate surfaces [26,27,28]. In addition, ZnO NRAs allow the loading of more functional sensing molecules due to their enlarged special surface area, which could potentially improve the sensing capability of the biosensors. Hence, it is essential to explore the feasibility of LAPS using ZnO NRA deposits for the direct label-free detection of bacterial DNA. 

Inspired by this idea, a LAPS with ZnO NRA deposits, which is a typical FED with flexible gate electrodes, was used for the direct label-free electrostatic detection of negatively charged bacterial ssDNA molecules using their intrinsic molecular charges for the first time. The probe ssDNA molecules were covalently attached onto the LAPS surface via the silanization process. ZnO NRAs deposited on the LAPS surface could enlarge the surface area necessary for the probe ssDNA immobilization and provide three-dimensional (3D) sites for the probe ssDNA molecules to hybridize more effectively with the target ssDNA. The hybridization of the probe ssDNA with the target *E. coli* O157:H7 ssDNA was measured by recording the shifts in the LAPS photocurrent–voltage (*I–V*) curves. It is worth noting that the LAPS chips deposited with ZnO NRAs and bacterial ssDNA sequences used in this study only demonstrate the technical feasibility of this novel approach for direct bacterial ssDNA sensing.

## 2. Materials and Methods

### 2.1. Fabrication and Functionalization of LAPS Chip

A LAPS chip was fabricated using a p-doped silicon wafer with a thickness of 400 µm (<100>, 1–10 Ωcm). A 30 nm SiO_2_ layer was developed on the surface of the silicon wafer through thermal oxidation. To create an Ohmic contact and light illumination, a 300 nm Al layer patterned with a window was fabricated on the rear side of the silicon wafer after etching the rear side of the SiO_2_ layer. The hydrothermal method was employed to prepare a layer of ZnO NRAs on the surface of the LAPS chip as previous reported [26]. Briefly, a thin layer of ZnO film was deposited on the surface of the LAPS chip via reactive magnetron sputtering, which was used as the seeding layer. The hydrothermal deposition was performed in a solution with equal volumes of Zn(NO_3_)_2_ and methenamine at the same concentration of 0.02 M in a sealed beaker. The surface on which the ZnO NRAs were expected to grow was put downward at 95 °C for 2 h. Then, the LAPS chip was thoroughly rinsed with deionized water, and blown dry with N_2_ for structure characterization and further sensing experiments.

The sequence of the probe ssDNA was designed specifically for the *E. coli* O157:H7 *eaeA* gene, which is a 30-base oligonucleotide (5’-AACGC CGATA CCATT ACTTA TACCG CGACG-3’). The sequences of the fully mismatched ssDNA were 5’-GCAGC GCCAT ATTCA TTACC ATAGC CGCAA-3’, which contains a fully mismatched base sequence to the probe ssDNA. Both the probe ssDNA and the fully mismatched ssDNA were synthesized by the Takara Biotechnology Company, Limited. To avoid any influence caused by solution changes on the measurement, 10 mM of phosphate-buffered saline (PBS) (pH 7.5) was used as the measurement solution for all the measurements and preparation of the probe ssDNA solutions. Next, 5′-end amino-modified probe ssDNA molecules were covalently immobilized on the LAPS surface via the silanization process. Briefly, the LAPS surface, deposited with ZnO NRAs, was treated with 0.1% (v/v) 3-aminopropyltriethoxysilane (APTES) in toluene with 1 h of incubation at room temperature (RT). Then, the LAPS surface was rinsed with the solvent and blown dry using N_2_, then heated for 2 h at 120 °C to solidify the attachment. The introduced amine residues reacted with the added glutaraldehyde overnight at room temperature. The introduced aldehyde residues reacted with the amine residues on the end of the probe ssDNA at RT for 12 h. Finally, a 1% bovine serum albumin (BSA) was added to block any unreacted aldehyde residues and any other non-specific binding sites on the ZnO NRAs. The sensor was rinsed with PBS and stored at 4 °C for further experiments.

### 2.2. Preparation of Target E. coli O157:H7 ssDNA

The target ssDNA with part of the base sequence complementary to the probe ssDNA was prepared from the *E. coli* O157:H7 *eaeA* gene using an asymmetric polymerase chain reaction (PCR). First, the *E. coli* O157:H7 were cultured in a nutrient broth at 37°C for 12 h, then it was killed using a 100 °C water bath for 15 min. Then, the *E. coli* O157:H7 was serially diluted to the desired concentrations with PBS by the surface plating-count method. The *E. coli* O157:H7 genomic DNA was extracted using an E.N.Z.A Bacterial DNA Kit (Beijing Solarbio Science & Technology Co. Ltd, China) following the product’s instructions and used as the DNA template for the asymmetric PCR in order to achieve the target *E. coli* O157:H7 ssDNA for label-free DNA detection, based on the hybridization of the target ssDNA with the probe ssDNA. The forward and reverse primers specific to the *E. coli* O157:H7 *eaeA* gene were 5’-GGCGG ATAAG ACTTC GGCTA-3’ and 5’-CGTTT TGGCA CTATT TGCCC-3’, respectively. For asymmetric PCR, the concentration of reverse primers was set to be 50 times higher than that of the forward primers. As a result, the forward primers played the role of a “limiting primer” (i.e., the target *E. coli* O157:H7 *eaeA* gene ssDNA will be generated by the reverse primer after the limiting forward primer was consumed). The asymmetric PCR reaction was carried out using a Bio-Rad Thermal Cycler (Bio-Rad Laboratories, Inc., Hercules, CA, USA) under the following conditions: 95 °C for 5 min preincubation, followed by 38 cycles of 30 sec denaturation at 95 °C, 30 sec of annealing at 55 °C, a 45 sec extension at 72 °C, and a 10 sec final extension. The product of the asymmetric PCR was a short ssDNA fragment (151 bases) of the *E. coli* O157:H7 *eaeA* gene, which contained the complementary base sequence to the probe ssDNA, thus allowing for the hybridization of the probe ssDNA and the target ssDNA on the LAPS surface for the detection of *E. coli* O157:H7 ssDNA. 

### 2.3. LAPS Measurement Setup and Target ssDNA Detection

**Figure 1:**
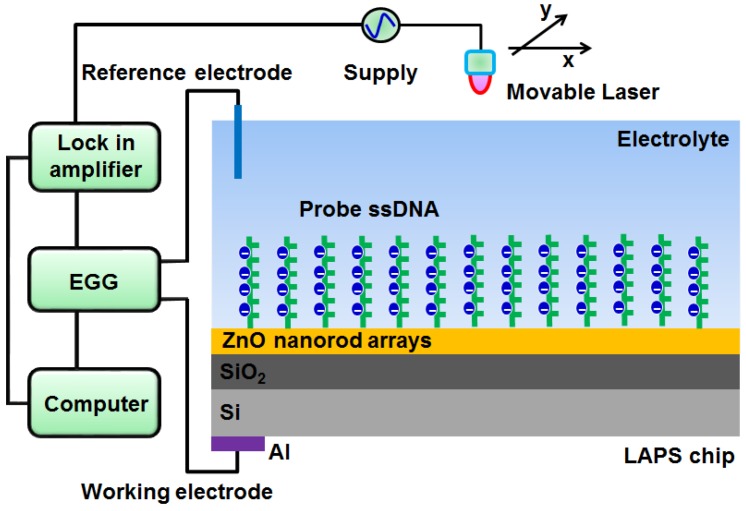
Schematic diagram of the light-addressable potentiometric sensor (LAPS) measurement setup.

Figure 1 is a schematic diagram of the LAPS measurement setup used in this study, which is similar to those in our previous reports [29]. The light source was an He–Ne semiconductor laser (Coherent Co., Santa Clara, CA, USA) with a wavelength of 543.5 nm and a diameter of 1 mm for illuminating the local area on the LAPS chip. A potentiostat (EG & G Princeton Applied Research, M273A, USA) was used to provide bias voltage; this was applied to the LAPS chip for the generation of photocurrent during illumination. A lock-in amplifier (model SR830 DSP, Stanford Research Systems) was utilized to amplify the photocurrent of the LAPS chip. A National Instruments data-acquisition card (DAQmx PCI-6259, National Instruments, TX, USA) was employed to record and collect the amplified photocurrent. Home-made LabVIEW software was used to control the whole measurement setup. All measurements were carried out at RT. The whole setup was shielded with a Faraday box to exclude ambient light and to minimize the influences of environmental factors on the measurements.

The LAPS surface charges changed after the probe ssDNA hybridized with the target ssDNA on its surface, which can be detected by recording the shifts of the LAPS photocurrent–voltage curves (*I–V* curves). First, the measurement solution (10 mM PBS, pH 7.5) was added to the detection chamber to record the *I–V* curves from the LAPS chip functionalized with the probe ssDNA. Then, the measurement solution in the detection chamber was removed and incubated with a solution containing the target *E. coli* O157:H7 ssDNA for 1 h at RT. Then, the measurement solution was used to wash the detection chamber, to remove any ssDNA molecules that did not hybridize with the probe ssDNA on the LAPS surface. Finally, the *I–V* curves were recorded from the LAPS surface after the DNA hybridization. The charge changes induced by the target ssDNA hybridization with the probe ssDNA on the LAPS surface can be indicated by a comparison of the *I–V* curves recorded from the LAPS before and after the target ssDNA hybridization. The shifts of the *I–V* curves were calculated and used as an indicator of the target ssDNA hybridization that occurred on the LAPS surface functionalized with the probe ssDNA.

## 3. Results and Discussion

### 3.1. Probe ssDNA Immobilization on the LAPS Surface

**Figure 2:**
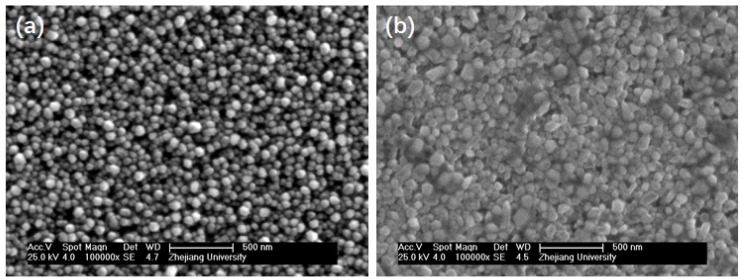
SEM images of ZnO nanorod arrays (NRAs) deposited on the LAPS surface (**a**) before and (**b**) after silanization with 3-aminopropyltriethoxysilane (APTES).

The surface of the LAPS chip used in this study was deposited with a layer of ZnO NRAs, which allowed us to load more probe ssDNA molecules due to their enlarged special surface area. A silanization process was performed to treat the LAPS surface and covalently immobilize the probe ssDNA on the sensor surface. A scanning electron microscope (SEM) was employed to characterize the ZnO NRAs deposited on the LAPS surface before and after silanization. Figure 2a shows the SEM image of the ZnO NRAs deposited on the LAPS surface before silanization. It can be observed that the orientation of the ZnO NRAs on the LAPS surface is highly ordered. The diameters of the ZnO NRAs are close to 50 nm, and their lengths and the space between the rods are about 500 nm and 100 nm, respectively. This geometry provided an ideal surface for the loading of more probe ssDNA. Figure 2b shows the SEM images of ZnO NRAs after silanization. It reveals that, with the treatment of 5% APTES, the attachment of APTES residues is apparent on the surface of the nanorods, thus providing sites for the further crosslink of the probe ssDNA using glutaraldehyde as bridges. Furthermore, after the silanization with APTES, the nanorods retained the same morphology, through which the probe ssDNA molecules could diffuse into and subsequently immobilize on the surface of the nanorods through a condensation reaction. 

**Figure 3:**
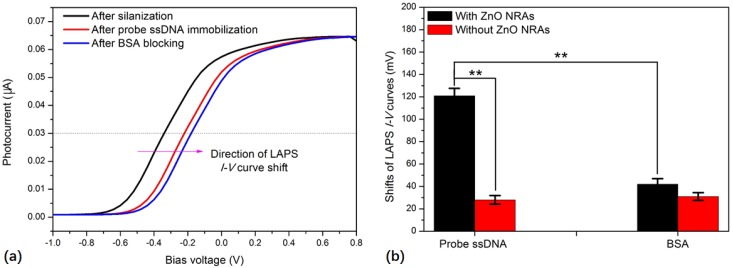
(**a**) *I–V* curves recorded from LAPS chip with ZnO NRAs after silanization, probe ssDNA immobilization, and bovine serum albumin (BSA) blocking. (**b**) Shifts of *I–V* curves induced by probe ssDNA immobilization and BSA blocking on the surface of LAPS chips with and without ZnO NRAs. All the data are represented by the mean ± standard error of the mean (SEM). ** *p* < 0.01, Student’s *t*-test. The mean and SEM of three experiments are shown.

For the detection of the target *E. coli* O157:H7 ssDNA, it was necessary to functionalize the LAPS surface with the probe ssDNA, which contained a specific complementary base sequence to the target ssDNA. BSA blocking is also desirable to avoid the non-specific adsorption of molecules on the sensor surface. The LAPS surface modifications mentioned above can lead to shifts in the LAPS *I–V* curves. It is due to the surface charge changes originated from the attachment of charged molecules on the LAPS surface. Figure 3a clearly shows that both the probe ssDNA immobilization and the BSA blocking-induced shifts of the LAPS *I–V* curves to the positive direction of bias voltage. This is mainly due to the adsorption of the negatively charged probe ssDNA and BSA molecules on to the LAPS surface. In addition, the optimal concentration of probe ssDNA was found to be 5 μM, as indicated by the highest shifts of the LAPS *I–V* curves. This reflects that the probe ssDNA molecules are immobilized on the LAPS surface with greater efficiency. As compared to the LAPS chip without the ZnO NRAs, significant higher shifts of the LAPS *I–V* curves can be induced by probe ssDNA immobilization (Figure 2b). This proves our hypothesis that the LAPS chip with ZnO NRAs is able to load more probe ssDNA. This could improve the capability of the sensing target ssDNA, especially in increasing the sensor dynamic range. On the other hand, with regard to the shifts of LAPS *I–V* curves induced by BSA blocking, no obvious difference was found between the LAPS chips with and without ZnO NRAs (Figure 2b). All the results demonstrated that, compared to the LAPS chip without ZnO NRAs, the LAPS chip with ZnO NRAs was able to load more probe ssDNA molecules, and this could potentially enhance the detection of the target ssDNA. 

### 3.2. Dectection of Target E. coli O157:H7 ssDNA

**Figure 4:**
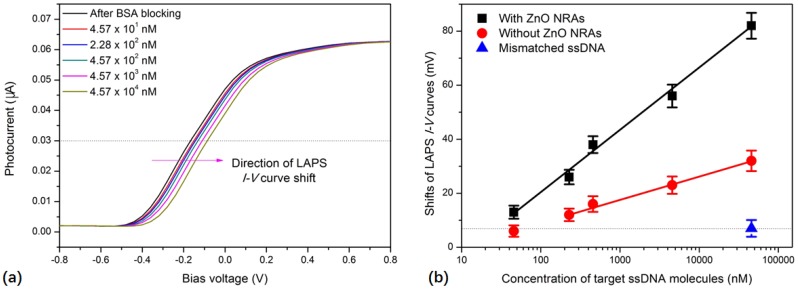
(**a**) Shifts of *I–V* curves recorded from the LAPS chip with ZnO NRAs in response to asymmetric polymerase chain reaction (PCR) products from different concentrations of *E. coli* O157:H7. (**b**) Statistical results of potential shifts of *I–V* curves recorded from LAPS chips with and without ZnO NRAs, induced by target ssDNA amplified from different concentrations of *E. coli* O157:H7. All the data are represented by the mean ± standard error of the mean (SEM). The mean and SEM of six experiments are shown.

After the probe ssDNA immobilization and BSA blocking, the prepared DNA biosensor was utilized for the detection of the target ssDNA amplified using an asymmetric PCR from different concentrations of *E. coli* O157:H7, ranging from 10 CFU/mL to 10^5^ CFU/mL. Asymmetric PCR makes the PCR product become ssDNA, which makes it possible to hybridize it with the probe ssDNA, thus allowing it to be detected directly by the DNA biosensor. Moreover, the DNA concentrations of asymmetric PCR products are proportional to the concentrations of *E. coli* O157:H7. The proportional constant between the concentrations of target DNA molecules and *E. coli* O157:H7 was estimated to be 2.75 × 10^11^ according to the thermal cycles used in the protocol of asymmetric PCR. Therefore, the target ssDNA concentrations corresponding to 10 CFU/mL and 10^5^ CFU/mL *E. coli* O157:H7 were estimated to be 4.57 nM and 45.7 μM, respectively. The measurement results indicate that asymmetric PCR products lead to the shifting of LAPS *I–V* curves to the more positive direction of bias voltage (Figure 4a). This is mainly attributed to the hybridization of the target ssDNA molecules with the probe ssDNA immobilized on the LAPS surface, which introduces more negative charges to the sensor surface, due to the molecules’ intrinsic negative charges. In addition, more shifts of the LAPS *I–V* curves were observed to the positive direction of bias voltage when the asymmetric PCR products from higher concentrations of *E. coli* O157:H7 were applied for measurement. The shift of the LAPS *I–V* curves caused by the asymmetric PCR products from concentrations lower than 100 CFU/mL of *E. coli* O157:H7 were negligible, which indicates that the detection limit of this DNA biosensor for the detection of *E. coli* O157:H7 was as low as 100 CFU/mL. 

The LAPS chip without ZnO NRAs was functionalized with the probe ssDNA and employed as a comparison to show the influences of ZnO NRAs on the performance of this DNA biosensor. As shown by the comparison in Figure 4b, it is obvious that the LAPS chip with ZnO NRAs showed a higher detection capability for *E. coli* O157:H7, as indicated by the higher slope of responsive linear curves. The detection limit of the LAPS chip without ZnO NRAs is 500 CFU/mL of *E. coli* O157:H7. This is probably because more probe ssDNA was attached to the LAPS chip with ZnO NRAs compared to the LAPS chip without ZnO NRAs. In addition, ZnO NRAs could provide 3D sites for the probe ssDNA molecules to hybridize with the target ssDNA more effectively. On the other hand, fully mismatched ssDNA alone was applied to test the specificity of this DNA biosensor. The results show that the fully mismatched ssDNA only induced negligible shifts in the LAPS *I–V* curves. This demonstrated a good specificity for the detection of target ssDNA amplified from the *E. coli* O157:H7.

However, since this DNA biosensor can only respond to target ssDNA, it is necessary to amplify the target ssDNA from *E. coli* O157:H7 using asymmetric PCR, which makes it impossible for this biosensor to detect *E. coli* O157:H7 directly from real samples. In further work, we will focus on the integration of sample preparation and treatment unit in this DNA biosensor, which could allow for the direct detection of real samples of *E. coli* O157:H7.

## 4. Conclusions

In this study, we have demonstrated that, with ZnO NRAs deposited on the surface of a LAPS, the hybridization of the target ssDNA amplified from *E. coli* O157:H7—with the probe ssDNA immobilized on the ZnO NRAs—can be detected by the shift of the *I–V* curve on the LAPS readout. Compared to the LAPS chip without ZnO NRAs, the sensing capability of DNA biosensors using a LAPS chip with ZnO NRAs improved significantly, as indicated by the higher responsive signals and lower detection limit. This improvement is mainly attributed to the dimensional and sensing properties of ZnO NRAs. In the near future, we will focus on the direct detection of ssDNA without amplification by integrating functional units to this DNA biosensor, which has great potential for the development of portable instruments toward the in-field detection of bacteria. With its ability to detect the hybridization of DNA molecules and its light-addressable characteristics, LAPS is a potential candidate for a new kind of label-free addressable DNA microarray and DNA chip, which has the advantages of saving time and decreasing the cost of potential applications in many fields, such as biomedicine, food and water quality control, and individualized medicine.
